# NLP-Driven Analysis of Pneumothorax Incidence Following Central Venous Catheter Procedures: A Data-Driven Re-Evaluation of Routine Imaging in Value-Based Medicine

**DOI:** 10.3390/diagnostics14242792

**Published:** 2024-12-12

**Authors:** Martin Breitwieser, Vanessa Moore, Teresa Wiesner, Florian Wichlas, Christian Deininger

**Affiliations:** Department for Orthopedic Surgery and Traumatology, Paracelsus Medical University, 5020 Salzburg, Austria; v.moore@salk.at (V.M.); f.wichlas@salk.at (F.W.); c.deininger@salk.at (C.D.)

**Keywords:** pneumothorax, CVC, central venous catheterization, removal, NLP, natural language processing, machine learning, value-based medicine

## Abstract

**Background**: This study presents a systematic approach using a natural language processing (NLP) algorithm to assess the necessity of routine imaging after central venous catheter (CVC) placement and removal. With pneumothorax being a key complication of CVC procedures, this research aims to provide evidence-based recommendations for optimizing imaging protocols and minimizing unnecessary imaging risks. **Methods:** We analyzed electronic health records from four university hospitals in Salzburg, Austria, focusing on X-rays performed between 2012 and 2021 following CVC procedures. A custom-built NLP algorithm identified cases of pneumothorax from radiologists’ reports and clinician requests, while excluding cases with contraindications such as chest injuries, prior pneumothorax, or missing data. Chi-square tests were used to compare pneumothorax rates between CVC insertion and removal, and multivariate logistic regression identified risk factors, with a focus on age and gender. **Results**: This study analyzed 17,175 cases of patients aged 18 and older, with 95.4% involving CVC insertion and 4.6% involving CVC removal. Pneumothorax was observed in 106 cases post-insertion (1.3%) and in 3 cases post-removal (0.02%), with no statistically significant difference between procedures (*p* = 0.5025). The NLP algorithm achieved an accuracy of 93%, with a sensitivity of 97.9%, a specificity of 87.9%, and an area under the ROC curve (AUC) of 0.9283. **Conclusions**: The findings indicate no significant difference in pneumothorax incidence between CVC insertion and removal, supporting existing recommendations against routine imaging post-removal for asymptomatic patients and suggesting that routine imaging after CVC insertion may also be unnecessary in similar cases. This study demonstrates how advanced NLP techniques can support value-based medicine by enhancing clinical decision making and optimizing resources.

## 1. Introduction

Central venous catheter (CVC) insertion is essential in modern medicine, particularly within critical care and emergency settings, offering vascular access for blood draws, medication administration, and parenteral nutrition [1,2,3]. It is particularly valuable during high-risk surgeries involving significant fluid shifts or blood loss and is commonly used for central venous pressure monitoring [4]. As populations age, disease severity increases, and comorbidity rates rise, the use of CVCs has surged in recent years [2,5]. However, contraindications such as severe coagulopathy, patient non-compliance, or local trauma at the insertion site must be carefully considered.

Despite careful assessment of contraindications, CVCs carry a notable risk of complications, with overall rates ranging from 1.1% to 19% [1,2,6,7]. Mechanical complications, particularly during insertion, pose significant risks to patient safety, with pneumothorax, hemothorax, and arterial puncture representing the most serious threats [1,2,7,8]. These mechanical issues occur either during the procedure or as a result of the catheter itself, requiring immediate attention to prevent life-threatening outcomes [9].

CVCs may be inserted into several anatomical sites, with the internal jugular vein (IJV), subclavian vein (SCV), and femoral vein being most commonly used. Each site offers distinct advantages and disadvantages that impact clinical decision making. This study focuses on the IJV and SCV due to their relevance in evaluating the incidence of pneumothorax following CVC procedures.

The IJV is often preferred for its straightforward access and lower risk of pneumothorax compared to the SCV, particularly when larger gauge needles are used or multiple insertion attempts are made [10]. However, the IJV may have a higher incidence of catheter-related infections, which is why the SCV is preferred in many clinical scenarios due to its lower risk of infection and thrombosis, making it more suitable for long-term access [1,11]. Understanding the risk profiles associated with these different insertion sites is crucial for optimizing patient outcomes and minimizing complications related to CVC placement [1,10,11].

Pneumothorax incidence during CVC insertion varies widely across sites, with reported rates between 0.2% and 6.6%. This variability depends on several other factors as well, including patient-related factors (e.g., COPD, abnormal anatomy, and prior trauma), catheter type, and clinical factors (e.g., physician experience, prior catheterizations, and emergency settings) [5,7,12,13].

Given the risk of serious complications, particularly pneumothorax, routine chest X-rays have traditionally been performed following CVC insertion to identify post-procedural issues. However, this practice is increasingly being questioned due to concerns over unnecessary costs, radiation exposure, and potential delays in care [13,14]. Moreover, post-procedural X-rays can provide a false sense of security, as radiographs, particularly in supine patients, may miss early signs of pneumothorax [14,15].

In contrast, routine chest X-rays after CVC removal are rarely performed, as the risk of mechanical complications, such as pneumothorax, is considered negligible. However, CVC removal can still result in rare but life-threatening complications, which may be overlooked due to their rarity. Among these, massive hemothorax and air embolism underscore the importance of continuous monitoring after CVC removal, as early detection and management are crucial in preventing fatal outcomes [16]. Proper procedural measures—such as keeping the patient supine and applying firm pressure at the insertion site—are essential to minimize these risks [17].

Despite the rarity of these complications, some hospitals still perform post-procedural imaging to rule out such events, even though international guidelines advise against routine imaging due to its limited clinical benefit [18]. This inconsistency emphasizes the importance of evidence-based practices in guiding CVC removal protocols.

At the hospital where this study was conducted, the routine use of chest X-rays after both CVC insertion and removal persists, despite guidelines advising against imaging in asymptomatic patients. This practice, driven by habit or institutional inertia, deviates from best practices and has resulted in the accumulation of a comprehensive database of electronic health records (EHRs) and radiology reports over many years. Rather than discarding these data, it presents an opportunity to assess real-world complication rates and the cost effectiveness of these practices, offering valuable insights for improving healthcare protocols and minimizing unnecessary interventions.

These EHRs are ubiquitous in medicine and critical to healthcare delivery, operations, and research. Despite their potential, EHRs are notoriously difficult to process, as over half of the information they contain is in the form of unstructured text (e.g., radiology reports, clinician requests, provider notes, and operation reports) and remains largely untapped for secondary use [19]. Recently, however, newer neural network and deep learning approaches to natural language processing (NLP) have made considerable advances, enabling the systematic analysis of such large datasets [20].

NLP, a branch of Artificial Intelligence (AI) focused on interpreting human language, allows for the efficient analysis of thousands of radiology reports, identifying key diagnoses like pneumothorax [21]. These AI-driven tools sift through extensive data, filtering out irrelevant information and streamlining processes with greater accuracy [22]. This approach transforms previously overlooked or underutilized data into actionable insights, revealing patterns that can inform clinical decision making and optimize resource use in line with evidence-based guidelines.

As EHRs become increasingly integrated with these AI tools, hospitals are now able to mine vast datasets to uncover trends and insights that would be difficult to identify manually. Recent advances in NLP, particularly with deep learning techniques, have been pivotal in automating the annotation of medical reports and transforming unstructured EHR data into actionable insights [23]. NLP plays an indispensable role in healthcare, with applications ranging from text classification to language translation and speech recognition [19]. As digital data continue to grow, NLP has become a cornerstone technology, enabling faster, more accurate decision making and better-informed clinical practices [20].

However, NLP models often face challenges when dealing with complex, institution-specific language in radiology reports. These reports often contain lengthy narratives, institution-specific abbreviations, and a mixture of normal and abnormal findings [23,24]. This complexity can hinder the accurate identification of specific conditions like pneumothorax, since such models tend to prioritize commonly reported normal results [25]. To overcome these challenges, advanced NLP techniques, such as word embeddings, deep learning, and transformer models like BERT (Bidirectional Encoder Representations from Transformers), developed by Google (Mountain View, CA, USA) have significantly improved the accuracy and efficiency of medical report analysis by enhancing the model’s ability to understand semantic context [26,27,28,29,30]. These innovations hold great promise for improving the precision and reliability of NLP applications in medical diagnostics [31].

By eliminating unnecessary post-CVC imaging and leveraging AI-driven tools like NLP, hospitals can improve the efficiency and cost effectiveness of healthcare delivery. This approach aligns with the principles of value-based medicine, which prioritize evidence-based, patient-centered care by reducing unnecessary interventions without compromising patient safety [32]. As healthcare shifts towards this model, technologies like NLP play a crucial role in optimizing both clinical outcomes and resource management, ensuring that interventions are guided by actual clinical benefits and not by habitual practices.

By developing an NLP algorithm specifically tailored to the language and abbreviations used at this institution, the study harnesses a large dataset of electronic health records to generate evidence-based recommendations for optimizing CVC-related practices. This approach has the potential to reduce unnecessary healthcare costs and patient exposure to radiation by re-evaluating the necessity of routine post-procedural imaging.

## 2. Materials and Methods

### 2.1. Study Design and Data Collection

This retrospective cohort study was conducted at the Salzburger Landeskliniken, a network of four university hospitals in Salzburg, Austria. This study focused on analyzing chest X-rays performed between 1 January 2012 and 31 December 2021 following CVC placement or removal. Data were collected from EHRs, and the primary aim was to evaluate the incidence of pneumothorax following CVC procedures.

The initial dataset, retrieved from the EHR system using specific keywords related to CVC procedures, consisted of 26,109 records. Each entry included patient demographic information (age and gender), the X-ray date, the department of admission, the radiologist’s report, and the clinician’s request for the X-ray. Sensitive patient information was de-identified, and the dataset was securely stored following institutional ethical guidelines approved by the Salzburger Ethics Committee.

The inclusion criteria were as follows:

- Chest X-rays conducted within 12 h of CVC placement or removal;
- Patients aged 18 years or older.
- The exclusion criteria were as follows:
- Patients under 18 years old;
- Incorrect CVC placement;
- Follow-up X-rays unrelated to the study objectives;
- Missing or incomplete data.

After applying these criteria, the final dataset included 17,175 patient records, with ages ranging from 18 to 101 years.

### 2.2. NLP Algorithm Development

A custom NLP algorithm was developed in R (Version 4.3.1) to detect new pneumothorax cases in radiology reports and clinician requests, while identifying contraindications such as chest injuries, recent surgeries, or previously known pneumothorax. The key methodological contributions of the algorithm are outlined as follows:

- **Custom phrase detection:** Clinicians manually identified common phrases and keywords related to pneumothorax, and regular expressions were created to capture these terms, including institutional abbreviations and common misspellings.
- **Text preprocessing**: Reports were preprocessed by converting them to lowercase, removing punctuation, and applying custom stopwords. A document–term matrix (DTM) was created with bi-grams (*n* = 2) using the tm package (Version 0.7-8) and RWeka package (Version 0.4-46) in R. Bi-grams were defined as consecutive pairs of words in the text, with two words in each token.
- **Feature selection:** Terms appearing in fewer than 1% of cases were excluded to focus on relevant patterns. This can be expressed as removing terms with a term frequency $f\left(t\right)$ such that
  $$f\left(t\right)\leq 0.01\times Ν$$
  where N is the total number of documents in the corpus.
- **Class imbalance handling:** Synthetic minority oversampling (SMOTE) was applied using the themis package (Version 1.0.2) to address class imbalance in pneumothorax cases. SMOTE works by generating synthetic examples for the minority class through interpolation. The interpolation formula for SMOTE is
  $$x_{new}=x_{minority}+\lambda \times (x_{neighbor}-x_{minority})$$
  where *x_new_* is the synthetic sample, *x_minority_* is the original minority class sample, *x_neighbor_* is the nearest neighbor, and *λ* is a random value between 0 and 1.
- **Model training:** The DTM was split into training and test sets (80/20 split), and a random forest model was trained on the balanced dataset with 250 trees and 2 predictors per split (mtry = 2). The random forest algorithm trains an ensemble of decision trees, where the number of trees T and the number of predictors per split m are key parameters. The training process minimizes the Gini impurity function for each split:
  $$Gini\left(t\right)=1-\sum_{i=1}^{C}p_{i}^{2}$$
  where *p_i_* is the proportion of samples belonging to class *i* in node *t*, and *C* is the number of classes.
- **Iterative refinement:** The algorithm was iteratively refined through manual reviews by a team of clinicians, improving detection accuracy through adjustments to expressions and model parameters.

The finalized NLP algorithm was applied to the 17,175 records to identify cases of newly discovered pneumothorax within 12 h of CVC procedures, taking into account the absence of contraindications. The primary outcome was the identification of pneumothorax in the radiology report or clinician’s request.

### 2.3. Ablation Study

We performed a broader ablation study to assess the impact of specific preprocessing steps and model configurations on the performance of our NLP algorithm for pneumothorax detection in radiology reports. To assess the impact of each component, we sequentially removed the following key preprocessing steps:

- **Removing custom stopwords:** We tested the model’s performance with and without custom stopwords derived from manual input to determine the relevance of this step in improving accuracy and specificity.
- **Evaluating different feature extraction methods:** We compared the use of tri-grams versus bi-grams to identify the most effective approach for capturing relevant contextual patterns in the text data.
- **Class balancing with SMOTE:** We evaluated models with and without SMOTE balancing to understand its role in managing class imbalance. This study highlighted that removing SMOTE significantly reduced sensitivity by 15%, underscoring the importance of this balancing technique.
- **Adjusting model hyperparameters:** We experimented with different random forest configurations, including variations in the number of trees (n_tree_ = 100, 150, 200, 250, and 300) and the number of predictors per split, to determine the optimal settings for balancing sensitivity and specificity.

This ablation study enabled us to optimize model parameters, refine preprocessing steps, and enhance the model’s accuracy for pneumothorax detection, while minimizing false positives and negatives.

### 2.4. Statistical Methods

Descriptive statistics were generated to summarize patient demographics and outcomes. Chi-square tests were conducted to compare pneumothorax rates between CVC insertion and removal procedures. Multivariate logistic regression was used to identify risk factors for pneumothorax, focusing on variables such as age and gender. All statistical analyses were performed using RStudio (Version 2024.04.2) and Microsoft Excel 2016.

Ethical approval for this study was granted by the Salzburger Ethics Committee, ensuring compliance with ethical standards and patient confidentiality on 30 March 2022 (ethical approval code 1032/2022).

## 3. Results

### 3.1. Patient Demographics

This study analyzed 17,175 CVC procedures conducted from 1 January 2012 to 31 December 2021. The patients’ ages ranged from 18 to 101 years (mean: 66.64 years, median: 69 years), with 9364 male patients (54.5%) and 7811 female patients (45.5%). Chest X-rays (CXR) were ordered from various clinical departments, with 9027 cases coming from internal medicine and smaller numbers from other specialties (Table 1).

Out of all of the procedures, 16,380 (95.4%) were CVC insertions, while 795 (4.6%) were CVC removals. CVC insertion durations ranged from 1 to 63 days, with a mean of 11.46 days and a median of 9 days.

### 3.2. Incidence of Pneumothorax

**Figure 1:**
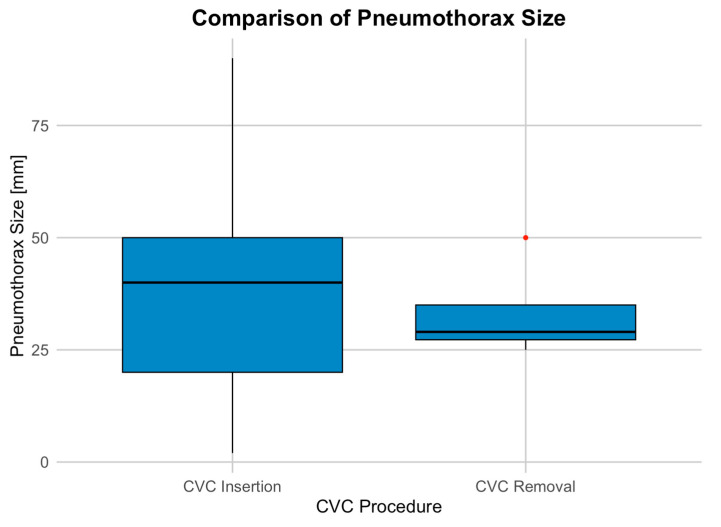
Distribution of pneumothorax sizes associated with CVC procedures.

Of the total cases, pneumothorax occurred in 109 instances (0.6%), with 106 (1.3%) following CVC insertion and only 3 (0.02%) after removal. The size of the pneumothoraces varied between 2 mm and 140 mm, with a mean size of 36.93 mm and a median size of 40 mm (Figure 1).

To assess the effect of CVC removal on pneumothorax size, a linear regression model was used. The logistic model examined the relationship between pneumothorax size and whether the CVC was removed. The intercept (37.139) suggests an average pneumothorax size of 37 mm when CVC removal is not considered. CVC removal had a non-significant effect, with a coefficient of −3.889, indicating a slight reduction in size (*p* = 0.713).

### 3.3. Statistical Analysis

#### 3.3.1. Chi-Square Test

A chi-square test was conducted to compare the incidence of pneumothorax between CVC insertion and removal. The results are presented in the contingency table below (Table 2).

The test yielded a chi-square value of 0.44965, with 1 degree of freedom and a *p*-value of 0.5025. These results suggest no statistically significant difference in pneumothorax incidence between CVC insertion and removal.

#### 3.3.2. Logistic Regression for Age and Gender

To evaluate the impact of age and gender on pneumothorax risk, a logistic regression model was used. The model fit statistics included a null deviance of 1300.1 and a residual deviance of 1295.7 with an AIC of 1301.7.

The results indicate a slight but statistically significant decrease in pneumothorax risk with increasing age (*p* = 0.0492), while gender was not a significant predictor (*p* = 0.3024) (Table 3).

### 3.4. NLP Algorithm Model and Performance

The final model, utilizing bi-grams with SMOTE balancing and 250 trees, achieved an accuracy of 96.6%, a sensitivity of 98.6%, and a specificity of 94.5%, as confirmed by manual review. Additionally, the model demonstrated an F1-score of 96.5%, indicating robust performance in accurately identifying pneumothorax cases while balancing false positives and negatives.

**Figure 2:**
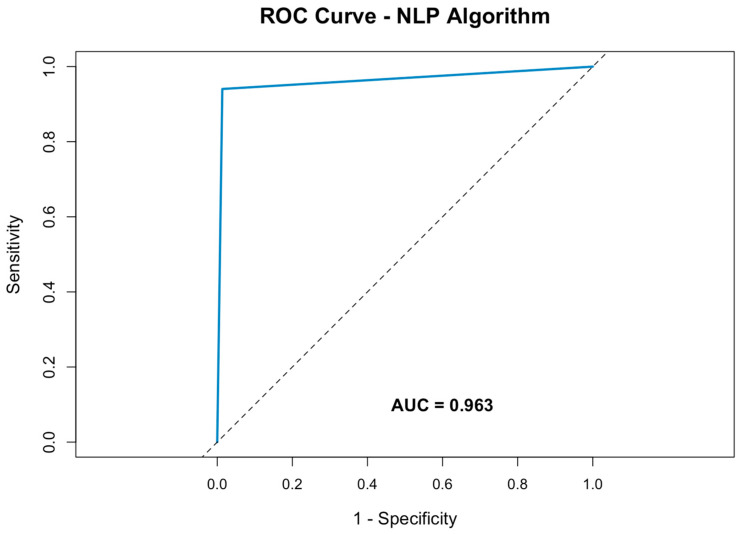
Receiver operating characteristic curve of the NLP algorithm for pneumothorax detection within radiology reports.

The performance of the NLP algorithm was further evaluated using a receiver operating characteristic (ROC) curve. The area under the curve (AUC) was calculated to be 0.9283, reflecting the algorithm’s strong ability to distinguish between pneumothorax-positive and pneumothorax-negative cases (Figure 2).

This demonstrates the model’s high discriminatory power, with an optimal balance between detecting true positives while minimizing false positives.

A comprehensive ablation study was conducted to examine the effects of various preprocessing steps, tokenization schemes, and model hyperparameters on the performance of the NLP algorithm developed to detect pneumothorax in radiology reports. Removing custom stopwords resulted in a drop in both accuracy (92.9%) and specificity (88.0%), indicating the importance of tailored vocabulary handling. Using tri-grams instead of bi-grams further reduced the accuracy to 87.7%, sensitivity to 98.1%, and specificity to 77.0%, suggesting that the bi-grams captured relevant contextual information more effectively for this task.

Omitting SMOTE balancing led to an increased accuracy of 98.6% but at the cost of a sensitivity of 0%, reflecting severe imbalance issues and demonstrating the necessity of balancing to ensure consistent pneumothorax detection. Testing different tree counts (100, 150, 200, and 300) in the random forest model yielded minor performance variations, with optimal results achieved at 250 trees. This model configuration provided the best balance between sensitivity and specificity, affirming it as the most effective combination for the dataset and task requirements.

This NLP algorithm was benchmarked against the ChatGPT-4 based detection model, developed by OpenAI (San Francisco, CA, USA), which showed an accuracy of 73.3%, a sensitivity of 62.0%, and a specificity of 73.4%. In comparison, the developed NLP algorithm outperformed the ChatGPT-4 model across all metrics, achieving a high balance between sensitivity and specificity, crucial for clinical applications where false negatives could lead to missed diagnoses. By utilizing custom keyword recognition, tailored preprocessing, and balanced model training, the NLP algorithm demonstrated superior robustness and accuracy in detecting pneumothorax cases from radiology reports. This comparison emphasizes the value of model customization and domain-specific training for sensitive medical applications.

## 4. Discussion

This study highlights the potential of customized NLP models for detecting pneumothorax in radiology reports following CVC procedures across large datasets, offering valuable insights into the possible optimization of imaging protocols and clinical decision making. Our findings demonstrate the value of machine learning-based approaches for scientific research, highlighting the efficiency these models bring in processing vast amounts of data. By automating the extraction and analysis of critical information, NLP algorithms can significantly reduce the time and effort required for manual review, making it possible to analyze thousands of reports in a fraction of the time. This not only accelerates research but also allows clinicians and researchers to focus on more complex tasks, thereby improving workflow efficiency. The ability to quickly sift through large datasets enables more robust and comprehensive scientific inquiries, leading to faster, data-driven insights that can inform clinical practices and healthcare policies.

By fine-tuning our NLP model to the hospital’s database and terminology, we achieved higher sensitivity and specificity in analyzing radiology reports related to CVC procedures. This approach is in line with the latest research, which highlights the importance of machine learning in improving NLP performance [33].

The algorithm demonstrated robust performance, with an accuracy of 93%, a sensitivity of 97.9%, and a specificity of 87.9%, alongside an AUC of 0.9283. Navarro et al. reviewed 94 studies, with 30 published in the last three years, highlighting that machine learning methods were employed in 68 studies, while 22 used a combination of machine learning and rule-based approaches. The highest F1-scores among these studies reached 93.25%, which compares favorably to the performance of our model [34].

Notably, while many studies rely on public datasets like i2b2, developed by Partners HealthCare (Boston, MA, USA), the proprietary dataset and manual review used in this study emphasize the importance of customization for specific clinical tasks, such as pneumothorax detection. By implementing NLP pipelines that are both precise and practical, hospitals can enhance clinical decision-making, minimize unnecessary interventions, and ultimately improve patient care. However, even though NLP applications in radiology have become more accurate, there is still a need for better clinical integration to fit seamlessly into real-world medical workflows [35]. This underscores the growing role of NLP in medical diagnostics and reinforces that adapting algorithms to specialized clinical contexts can lead to improved outcomes, rather than solely relying on generalized public datasets.

When benchmarking the performance of our custom-built NLP algorithm against a ChatGPT-4o-based detection model, we observed a significant disparity in performance. The ChatGPT-4o model demonstrated an accuracy of 73.3%, a sensitivity of 62.0%, and a specificity of 73.4%. Therefore, our model outperformed ChatGPT-4o across all metrics, and its high balance between sensitivity and specificity is crucial in clinical settings, particularly for conditions like pneumothorax, where false negatives could lead to missed diagnoses and serious patient outcomes. The use of custom keyword recognition, tailored preprocessing (e.g., institution-specific terms and abbreviations), and balanced model training significantly contributed to the robustness of our algorithm. These features enabled the model to capture context-specific patterns in radiology reports more effectively than generalized models like ChatGPT-4o.

The ChatGPT-4-based approach, while powerful for general language processing tasks, reflects the limitations of generalized language models in high-stakes clinical settings [25]. The ability of these models to effectively handle clinical language, which often includes jargon, abbreviations, and specific terminology, is limited. For clinical applications like pneumothorax detection, where precision and recall are paramount, domain-specific training becomes essential. In future work, hybrid models that combine pre-trained language models with extensive domain-specific customization could improve performance, further enhancing the sensitivity and specificity of NLP algorithms in medical diagnostics. Such an approach could bridge the gap between general-purpose models and the specialized needs of clinical applications, offering a more adaptable and efficient solution.

One of the primary goals of this model-based study was to evaluate the necessity of routine post-procedural chest X-rays, a practice that is often questioned due to concerns about unnecessary costs, radiation exposure, and potential delays in care.

While the rate of pneumothorax following CVC insertion and removal is low, with 1.3% of patients developing pneumothorax after CVC insertion and only 0.02% after removal, our analysis did not find any statistically significant difference between the two groups (*p* = 0.5025). Based on these findings, we cannot definitively recommend against routine imaging after CVC placement, especially in symptomatic patients, but it is clear that routine imaging for asymptomatic patients may not provide substantial clinical benefits.

Our findings align with existing literature that questions the need for routine chest X-rays following CVC procedures [15]. While complications such as arterial puncture, hematoma and infection remain concerns—with incidence rates of 2% for arterial puncture, 4% for hematoma, and 9% for sepsis—the rates of pneumothorax and catheter malposition are low enough to suggest that routine post-procedural imaging may be unnecessary for asymptomatic patients [36]. Recent studies report pneumothorax rates ranging from 0.2% to 6.6% after CVC insertion, reinforcing the notion that routine chest radiographs may not be justified given the low complication incidence [5,7,12,13].

Although chest X-rays have traditionally been the standard for detecting pneumothorax post-CVC, recent studies indicate that ultrasound offers a more accurate, immediate, and cost-effective alternative [15]. Alrajhi et al. demonstrated that ultrasound has a sensitivity of 90.9% and specificity of 98.2% for pneumothorax detection, far surpassing chest radiographs, which have a sensitivity of just 50.2% [9]. This significant disparity reveals that post-procedural X-rays may provide a false sense of security, as early signs of pneumothorax can easily be missed, particularly in supine patients during chest radiographs [14,15].

Despite these findings, the routine use of chest X-rays after CVC procedures continues in many clinical settings, often driven by institutional habits rather than evidence supporting a transition to ultrasound for pneumothorax detection [15]. The reluctance to adopt newer practices like ultrasound stems from several barriers, including the upfront costs of ultrasound machines, the resources required to train clinicians, and legal concerns [37]. Many institutions remain hesitant to adopt ultrasound, largely due to the perception that it requires extensive training compared to traditional imaging methods. However, data show that with proper instruction and supervision, ultrasound yields similar success rates in CVC placement and complication prevention as conventional methods [38,39].

Financial analyses have also indicated modest labor cost savings when ultrasound is used in emergency departments and intensive care units to confirm catheter positioning and exclude pneumothorax [39,40,41]. Institutional inertia also plays a significant role, as many clinicians rely on established practices, even in the absence of updated evidence [42]. Legal concerns exacerbate this inertia, with providers fearing potential repercussions for deviating from established protocols, even when newer, proven methods are available [15,37].

While physicians are generally well aware of the risks of catheter insertion, complications from CVC removal often receive less attention, despite the procedure carrying its own set of hazards. Inadequate training, particularly in removal techniques, has been linked to increased risks, including air embolism. Ensuring that only trained personnel handle CVC removal is key to reducing these potentially life-threatening complications [17]. Risks such as bleeding, catheter fracture, thrombus dislodgement, and infection, though rare, can lead to severe consequences if not properly managed [16]. Proper education and adherence to protocols are essential to prevent these events in this routine procedure.

To address these challenges within the framework of value-based healthcare, developing standardized protocols ensures that imaging is performed only when clinically necessary. This approach enhances patient care while reducing unnecessary radiation exposure and healthcare costs, aligning with value-based principles by prioritizing patient-centered, evidence-driven practices. By implementing clear guidelines that favor ultrasound over chest X-rays, hospitals can streamline patient management, promote more efficient care delivery, and reduce unwarranted interventions, thus contributing to both improved outcomes and resource optimization [26].

Despite the importance of our findings, several limitations must be considered, which may affect the generalizability and validity of the results, as well as areas that warrant further investigation. First, the dataset is restricted to chest X-rays containing specific search terms in the physician’s request, meaning that some X-rays for CVC insertion or removal may have been overlooked. Additionally, while the NLP algorithm was customized to the institution’s unique language and abbreviations, this limits its broader applicability to other settings. Moreover, the study primarily focuses on post-procedural pneumothorax, excluding other complications such as infections or catheter misplacement, which could provide a more comprehensive understanding of CVC-related risks.

A major gap in our study is the lack of detailed characterization of the pneumothorax cases identified. Although pneumothorax was found in a small percentage of the patient population (1.3% for CVC insertions and 0.02% for CVC removals), we did not provide detailed information on the size, location (e.g., apical vs. basal), or clinical context of these cases. The size and location of pneumothorax (e.g., apical pneumothorax, which may be more symptomatic) could play a crucial role in determining whether imaging is warranted.

Further characterization of pneumothorax, including its size, location, and symptoms, would provide a more comprehensive understanding of when imaging is truly necessary. Additionally, the hemodynamic status following pneumothorax—such as whether patients develop respiratory distress or cardiovascular instability—could further guide decision making regarding routine imaging. It would also be valuable to explore how the clinical situation at the time of CVC placement (e.g., patient comorbidities and type of surgical procedure) affects the risk of pneumothorax. This could lead to more precise risk stratification models, ensuring that routine imaging is reserved for high-risk cases, which would help reduce unnecessary healthcare costs and minimize patient exposure to radiation.

Given the low rates of pneumothorax observed in our study, it is important to consider that future research should investigate these factors further, including clinical outcomes and complications associated with pneumothorax of different sizes and locations. A more comprehensive evaluation could provide clearer guidance on when imaging after CVC insertion and removal is truly necessary, helping to streamline clinical practices and optimize the use of healthcare resources.

## 5. Conclusions

While the results of this study align with existing recommendations against routine imaging following CVC procedures for asymptomatic patients, the low rate of pneumothorax does not justify a complete abandonment of imaging practices.

However, additional data on the clinical implications of pneumothorax, including size, location, and patient characteristics, will be necessary to further optimize imaging protocols and improve patient outcomes in this area. This supports a shift towards more efficient, evidence-based practices in line with value-based healthcare principles.

This study underscores the effectiveness of tailored NLP algorithms for pneumothorax detection in radiology reports, significantly enhancing research efficiency and data management in electronic health records. By automating the extraction and analysis of key clinical information, NLP algorithms enable the rapid processing of vast datasets, which would be time-consuming and error-prone if performed manually. This not only reduces the workload of clinicians and researchers but also facilitates the identification of patterns and trends that may go unnoticed in manual reviews.

Moreover, tailored NLP algorithms, specifically designed for an institution’s unique language and terminology, provide more precise and reliable results compared to generic large language models (LLMs), which may struggle with medical jargon and context-specific abbreviations.

## Figures and Tables

**Table 1:** Distribution of CVC procedures across clinical departments.

Clinical Department	Number of Cases (%)
Internal medicine	9027 (52.6%)
Neurology	1869 (10.9%)
Surgery	1427 (8.3%)
Neurosurgery	884 (5.1%)
Trauma and orthopedics	1121 (6.5%)
Vascular surgery	682 (4.0%)
Other specialties	2165 (12.6%)

**Table 2:** Contingency table of pneumothorax incidence between CVC insertion and removal.

	No Pneumothorax	Pneumothorax
CVC insertion	16,274	106
CVC removal	792	3

**Table 3:** Logistic regression analysis of risk factors associated with pneumothorax.

Predictor	Estimate	Std. Error	Z-Value	*p*-Value
Intercept	−4.396298	0.398671	−11.027	<2 × 10^−16^
Age	−0.011809	0.006003	−1.967	0.0492
Gender	0.200964	0.194861	1.031	0.3024

## Data Availability

The data presented in this study are available upon request from the corresponding author due to ethical and privacy restrictions regarding patient information.
